# Effect of Mixed Reduction Approach on the Oil Absorption Capacity of Graphene Oxide Aerogels

**DOI:** 10.3390/ma19030632

**Published:** 2026-02-06

**Authors:** Carlos Cargua, Nelly Maria Rosas-Laverde, Arturo Barjola, Enrique Giménez, Alina Iuliana Pruna

**Affiliations:** 1 Department of Materials, Escuela Politécnica Nacional, Quito 170524, Ecuador; vinicio.cargua.l@gmail.com (C.C.); nelly.rosas@epn.edu.ec (N.M.R.-L.); 2 Instituto Universitario de Tecnología de Materiales, Universitat Politècnica de València (UPV), Camino de Vera s/n, 46022 Valencia, Spain; arbarrui@doctor.upv.es (A.B.); enrique.gimenez@mcm.upv.es (E.G.)

**Keywords:** graphene oxide, aerogels, oil absorption, reduction, freeze-casting, annealing

## Abstract

This study evaluates the impact of a comprehensive design integrating precursor type, reduction and freeze-casting on the development of aerogels with high sorption capacity for engine oil. In this respect, the graphene oxide was varied from commercial to expanded; the reduction approach relied either on purely hydrothermal or combined hydrothermal–chemical reduction approaches. Following the synthesis, freeze-casting was applied at −5 °C and −196 °C. To further improve the reduction degree, annealing in an inert atmosphere was employed upon drying. The effects of precursors, reduction approach, freeze-casting and annealing were systematically investigated. Characterization techniques, including FT-IR, Raman spectroscopy, SEM, and EDS, were used to correlate the degree of reduction and morphological features of the porous structure with the absorption properties. The use of expanded GO as a precursor yielded aerogels with more homogeneous three-dimensional networks, a reduced bulk density of 3 mg cm^−3^, and lower oxygen-containing functional group content, thereby achieving consistently superior oil absorption of 270 g g^−1^, with an oil occupancy of 94%. The process was found to fit well with the pseudo-first-order kinetic model. The results demonstrate that a comprehensive approach—considering combined reduction, freeze-casting, and thermal annealing—enables the tailored optimization of both the structure and absorption performance of GO aerogels for the remediation of oil spills.

## 1. Introduction

The presence of varying pollutants such as oils, dyes, antibiotics, microplastics, and their derivatives in aquatic environments constitutes a significant threat to the environment and the ecosystems within aquatic habitats [1,2]. It is imperative to mitigate these negative impacts by effectively removing such pollutants from aquatic systems [3,4,5]. Due to its ease of handling, high efficacy, cost-effectiveness, and environmental sustainability, physical absorption remains the most compelling and widely adopted method for contaminant removal [6,7,8]. Absorbent materials have been employed to remove dyes, heavy metals and organic solvents from aqueous media [9,10,11]. The predominant absorbents include zeolites, polyurethane foams, resins, fibers, biomass, and activated carbon [10,12]. The ideal characteristics of a sorbent include high hydrophobicity, oleophylicity, high porosity, and substantial absorption capacity [13].

Carbonaceous materials are regarded as excellent candidates for absorbent applications owing to their high specific surface area and superhydrophobic properties [14]. Amongst these, graphene oxide (GO) is a versatile two-dimensional material rich in oxygenated functional groups which enable surface modifications [15,16]. The π–π stacking interactions of GO facilitate bonding with organic compounds, leading to spontaneous absorption processes that are energetically favorable [5,15,16,17,18]. However, the two-dimensional nature of GO means it lacks porosity and exhibits a high tendency to agglomerate, which can negatively impact pollutant absorption efficiency [19]. On the other hand, three-dimensional (3D) GO structures are more advantageous due to their enhanced porosity, higher specific surface area, and improved structural stability, all of which promote more effective contaminant removal [20]. Thanks to the versatility in the surface chemistry of GO allowing for easy processing, GO materials are often preferred over other similar materials like carbon nanotubes for the synthesis of aerogel sorbents.

Numerous methodologies exist for the synthesis of 3D GO aerogels, including hydrothermal reduction, chemical reduction, cross-linking, sol–gel processes, and innovative 3D printing techniques [16,21,22]. To improve the absorption characteristics of GO aerogels, various surface modification techniques involving modifying agents, alteration of the porous structure, physical assembly procedures, selection of precursors, and post-synthesis treatments have been employed [23,24,25,26].

Freeze-casting is known for its direct effect on the pores of aerogel as the freezing rate influences the kinetics of water–ice structuring within the hydrogel [23]. Consequently, rapid freezing typically yields compact structures with a higher density of small pores, whereas slower freezing yields structures with larger, interconnected pores [23,27].

Removal of the oxygen-containing functional groups represents one of the most common approaches to control the hydrophobicity of GO aerogels. Chemical reduction employing varying agents such as vitamin C yields reduced GO (rGO) nanosheets that spontaneously cross-link to form a hydrogel, facilitated by π–π interactions, hydrophobic effects, H–H interactions, and Van der Waals forces [28,29]. Consequently, by increasing the reduction degree of GO, the C=C bonds are restored, and π–π hydrophobic interactions are promoted, thereby improving the absorption capacity.

However, it is noteworthy that the reduction of GO results in nanosheet stacking [24]. Therefore, a combined approach based on chemical reduction and freeze-casting is advised to obtain a 3D monolith with improved mechanical characteristics and good porosity and hydrophobicity [30,31]. The hydrophobicity enhancement by removal of oxygen-containing functional groups or functionalization of GO can be tailored by thermal annealing at elevated temperatures in an inert or vacuum-controlled atmosphere [32,33,34].

In this work, we present a combined approach for the design of reduced graphene oxide aerogels for engine oil absorption. To the best of our knowledge, most studies up until now have focused on only one or two approaches in the manufacturing process, and therefore, a comprehensive understanding of the combined effect on oil absorption performance is needed. The proposed methodology incorporates a balanced choice between precursor, reduction method, freeze-casting, and thermal annealing. Recognized for its simplicity, reproducibility, and potential scalability, this approach produces lightweight, hydrophobic aerogels with a robust, low-density 3D network and enhanced oil absorption capacity. The obtained results provide a foundational framework for precisely modulating the absorption properties of graphene oxide aerogels, thereby advancing their application for contaminant removal from aquatic environments.

## 2. Materials and Methods

### 2.1. Chemicals and Reagents

Graphene oxide (GO) dispersion (2 mg mL^−1^, commercial grade) was provided by Graphenea Tech Co. (Donostia, Spain). Expanded graphite (thermal, precursor material) was acquired from Avanzare S.L. (La Rioja, Spain) and employed in a typical Hummers synthesis to obtain expanded GO (GO_e_, dispersion provided by Graphenea Tech Co. (Donostia, Spain). L-ascorbic acid (vitamin C, reagent grade), concentrated sulfuric acid (H_2_SO_4_, 98%), potassium permanganate (KMnO_4_, ACS reagent, 99%), hydrogen peroxide (H_2_O_2_, 35%), potassium persulfate (K_2_S_2_O_8_, reagent grade, 99%), phosphoric acid (P_2_O_5_, reagent grade), and hydrochloric acid (HCl, 37%) were obtained from Sigma-Aldrich (Madrid, Spain). EDGE Turbo Diesel SAE 5W–40 engine oil was supplied by Castrol España, and red oil dye type O was acquired from Sigma-Aldrich (Madrid, Spain).

### 2.2. Preparation of Graphene Oxide Aerogels

The hydrogel was synthesized from graphene oxide dispersions (GO and GO_e_) by hydrothermal/chemical reduction procedures. Subsequently, it underwent freeze-casting and freeze-drying to remove ice crystals to generate reduced graphene oxide aerogel (denominated rGO or rGO_e_, depending on the precursor). Finally, it was thermally annealed in an inert atmosphere, the obtained monolith being denominated r_T_GO or r_T_GO_e_.

#### 2.2.1. Synthesis of GO Hydrogels

Dispersions of 2 mg mL^−1^ of GO were sonicated for 1 h to obtain an aqueous dispersion of GO. For hydrothermal reduction (denominated r_1_), the cylindrical containers with GO dispersions were placed in a hydrothermal reactor for 4 h at 140 °C to obtain a hydrogel [7,35]. The aerogels produced via this method were designated as r_1_GO and r_1_GO_e_, depending on the precursor [35]. The combined synthesis route (denominated r_2_) integrated hydrothermal conditions with the use of a reducing agent (vitamin C) in a 1:2.5 weight ratio of GO:Vitamin C [22]. This pathway provided the aerogels denominated as r_2_GO and r_2_GO_e_, respectively.

#### 2.2.2. Freeze-Casting of GO Hydrogels

Following the synthesis, the hydrogels were subjected to freeze-casting in either conventional freezers at −5 °C for 48 h or to instantaneous freezing at −196 °C in liquid nitrogen. The freeze-cast GO hydrogels were subsequently dried at 20 °C for 48 h under a vacuum pressure of 0.0015 mbar in a LyoQuest lyophilizer (Telstar, Madrid, Spain).

#### 2.2.3. Thermal Annealing of the Aerogel

To improve the removal of oxygen-containing functional groups, the obtained aerogels were annealed in a tubular furnace at 600 °C for 1 h, with a heating rate of 300 °C h^−1^ under nitrogen atmosphere.

### 2.3. Characterization

The morphology and elemental composition were determined using a field-emission scanning electron microscope (FE-SEM, Gemini SEM 500, Zeiss, Oberkochen, Germany) equipped with an energy-dispersive X-ray spectrometer (EDS) (Octane Plus, Zeiss, Oberkochen, Germany). The variation in oxygen content attributable to the degree of reduction was analyzed via Fourier transform infrared spectroscopy (FT-IR-62000, Jasco, Madrid, Spain) in attenuated total reflectance (ATR) mode across the spectral range of 400–4000 cm^−1^. The extent of graphitization, oxidation, and structural defects was evaluated employing Raman spectroscopy (Xplora, Madrid, Horiba, France) with 532 nm laser excitation. The apparent density of the aerogel was determined using the mass of the aerogel and its volume considered as a cylinder. The structure and composition of the aerogels was analyzed by X-ray Photoelectron Spectroscopy (XPS) using a VG-Microtech Multilab 3000 photoelectron spectrometer (Thermo Fisher Scientific Inc., Waltham, MA, USA). The C1s high-resolution peaks were deconvoluted with the help of CASAXPS 2.3.17 software (Casa Software Ltd., Wilmslow, Cheshire, UK) by applying a Shirley baseline subtraction and a Gaussian–Lorentzian (70%:30%) peak shape.

### 2.4. Absorption Measurements

The obtained aerogels were immersed in oil-containing distilled water. In order to exclude water sorption from the calculations, the absorption capacity was measured by recording absorption duration of subsequent increments of 20 μL oil added to the water. The total volume of absorbed oil was calculated as the sum of the oil increments absorbed over 10 min. The mass of the absorbed oil was computed considering the oil density and total volume of absorbed oil. The gravimetric absorption capacity was determined by dividing the total mass of absorbed oil (m_oil_) by the aerogel mass (m_A_)

The absorption rate (AR) was calculated according to Equation (1), where t is the absorption duration:

(1) $$AR=\frac{m_{oil}}{m_{A}}\times \frac{1}{t}\;\;\;\left[\frac{g\;g^{-1}}{min}\right]$$

Finally, the pore volume after filling with oil was calculated by dividing the absorbed oil volume by the pore volume, V_p_, where the latter represents the theoretical total pore volume as defined by Equation (2):

(2) $$V_{P}=m_{A}\left(\frac{1}{\rho_{A}}-\frac{1}{\rho_{G}}\right)\;$$

In this context, (ρ_A_) denotes the apparent density of the aerogel, whereas (ρ_G_) represents the density of graphite assumed as 2.2 g cm^−3^.

Equation (3) for the pseudo-first-order (PFO) model Equation (3),

(3) $$q\left(t\right)=q_{e}\left(1-e^{-k_{1}t}\right),$$

was used to analyze the kinetic data, where *q*(*t*) (mg g^−1^) is the amount of oil absorbed on the aerogel at any time t (h) and k_1_ (min^−1^) is the PFO kinetic model constant. The goodness of fit was assessed from R^2^ values using Origin 8.5 software.

## 3. Results

### 3.1. Synthesis and Characterization of Aerogels

**Figure 1:**
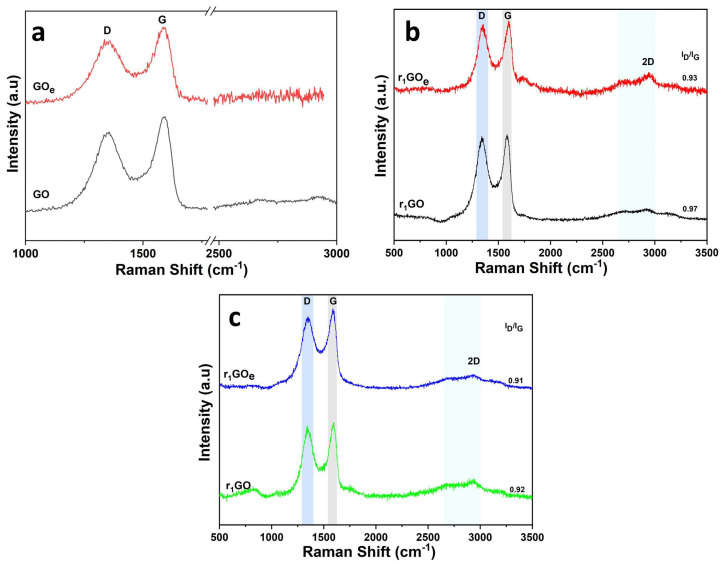
(**a**) Raman spectra (shaded typical bands D, G and 2D) of precursor dispersions; (**b**) aerogels obtained by hydrothermal reduction (r_1_) and freeze-casting at −5 °C; (**c**) aerogels obtained by hydrothermal reduction (r_1_) and freeze-casting at −196 °C.

**Figure 2:**
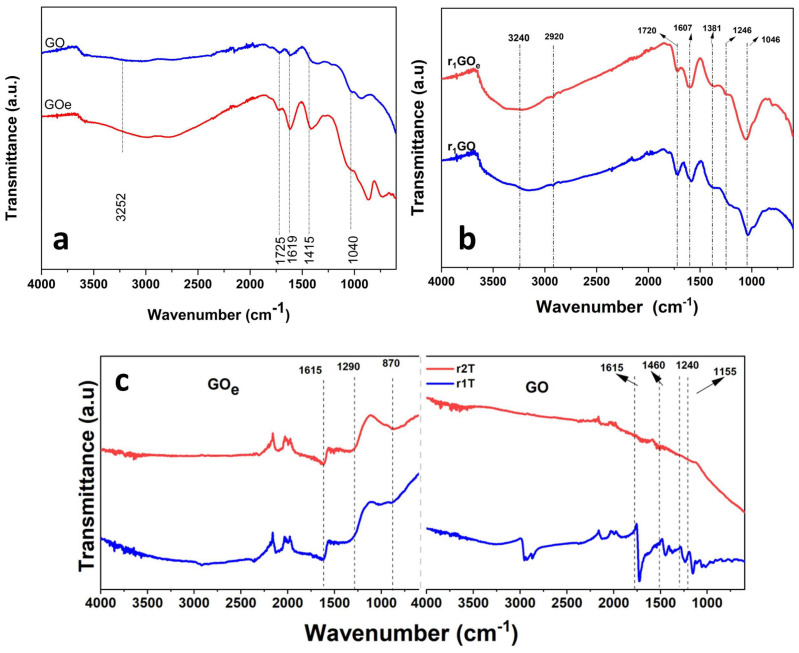
FT-IR spectra of precursors (**a**); aerogels obtained by hydrothermal reduction (r_1_) and freeze-casting at −196 °C (**b**); aerogels freeze-cast at −5 °C and further annealed (**c**).

The precursor significantly affects the reduction and assembly of aerogels, as shown below. The synthesis of GO aerogels was conducted using dispersions of commercial graphene oxide (GO) and an expanded graphite-based derivative (GO_e_). Raman spectra of the GO-based precursors are presented in Figure 1a. As observed, both precursors show characteristic D and G bands at 1349–1352 cm^−1^ and 1589–1591 cm^−1^ [28]. The D-to-G band intensity ratios (ID/IG) are 0.83 for GO and 0.85 for GO_e_, indicating a greater number of structural defects in GO_e_ due to oxygen-containing functional groups within the GO sheets [36]. As further shown in Figure 2b,c, the Raman spectra of the aerogels as a function of freeze-casting temperature reveal higher D/G band intensity ratios than those of their precursors, indicative of the removal of oxygen-containing functional groups.

On comparing the Raman spectra of the aerogels obtained by freeze-casting in a conventional freezer with those obtained via freeze-casting in liquid nitrogen, the D/G band intensity ratio appears lower for the aerogels obtained in liquid nitrogen, associated with more defects due to slower freezing rate. Moreover, the D/G band intensity ratio appears lower for the aerogels obtained from the GO_e_ precursor. This evolution can be explained by the fact that the D/G band intensity ratio doesn’t differentiate between holes and sp^3^ defects, instead indicating an increased number of smaller-sized sp^2^ domains resulting from the combined reduction approach and from aerogels obtained from GO_e_, respectively. The low 2D-to-G band intensity ratio (with the 2D band appearing at approximately 2700 cm^−1^) of approximately 0.1 indicates an aerogel with stacked sheets.

As shown in Figure 2a, the Fourier transform infrared (FT-IR) spectra of the precursors exhibit similar features, namely, C–H stretching vibrations at 2960 cm^−1^ and hydroxyl groups (O–H) associated with water molecules near 3585 cm^−1^. The carbonyl vibration C=O appeared at 1725 cm^−1^, the aromatic C=C band appeared at 1619 cm^−1^, the carboxylic vibration C–OOH was located at 1415 cm^−1^, and the alkoxy C–O vibration at 1040 cm^−1^ [37,38]. Notably, GO_e_ shows higher intensities of the carboxyl and hydroxyl bands, indicating an increase in defect sites due to improved oxidation of thermally expanded graphite.

Further, Figure 2b shows the FT-IR spectra of aerogels produced via hydrothermal synthesis and freeze-casting at −196 °C, which exhibit lower intensities of the bands corresponding to oxygen-containing groups than those of the precursors, namely the C=O (carbonyl), C–OOH (carboxyl), and C–O (epoxy or alkoxy) groups. These findings confirm a trend toward partial deoxygenation of graphene oxide during hydrothermal synthesis [16,36]. On the other hand, the backbone band C=C (graphene) increases in intensity upon reduction due to recovery of sp^2^ domains. Overall, the intensity levels are generally lower in r_1_GO than in r_1_GO_e_, while the C=C band appears stronger in r_1_GO_e_. Considering the use of freeze-casting at slower rate (−5 °C) as being the harshest conditions for the reduction of GO, the effect of thermal annealing on the corresponding aerogels is further depicted in Figure 2c (denominated as rxTGO or rxTGO_e_, where x refers to reduction via the hydrothermal method (1) and reduction via the hydrothermal–chemical approach (2)). The spectra corresponding to hydrogels freeze-cast at −5 °C and annealed showed the highest reduction in the intensity of bands associated with oxygenated functional groups, suggesting ongoing deoxygenation and partial restoration of the graphitic structure in the graphene oxide network, thereby increasing its hydrophobicity [16,39].

**Figure 3:**
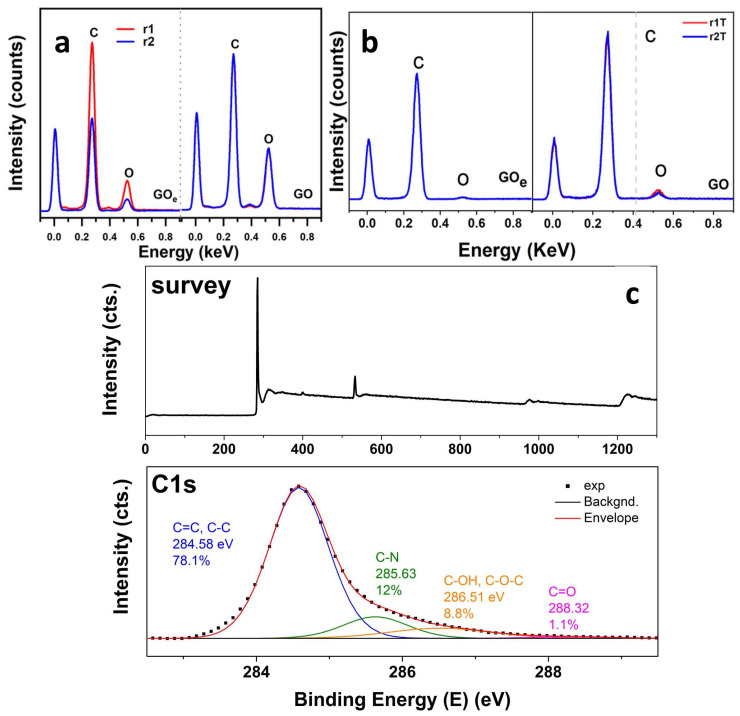
EDS spectra of the aerogels obtained by freeze-casting at −5 °C (**a**) before and (**b**) after thermal annealing (r_1_—hydrothermal reduction, r_2_—combined reduction, T—thermal annealing). (**c**) XPS survey and C1s spectra for aerogel obtained by hybrid reduction and thermal annealing.

As freeze-casting in liquid nitrogen produces rGO sheets with similar features in FT-IR and Raman spectra, the EDS spectra are further shown in Figure 3 for the aerogels obtained by freeze-casting in a conventional freezer, as being representative of the harshest conditions. The EDS analysis before the annealing treatment (Figure 3a) revealed an increased C/O ratio for aerogels obtained from the GO_e_ precursor, and when the combined reduction approach was applied, the C/O ratio increased further by almost 5-fold, indicating that the combined method was more effective at removing oxygenated functional groups than hydrothermal synthesis alone for an expanded GO. Upon applying thermal annealing (Figure 3b), the C/O ratio increased even further. For GO, both reduction methods resulted in similar C/O ratio. This was demonstrated for the aerogels obtained by freeze-casting at −5 °C, while in the case of GO_e_, the combined approach of hybrid reduction and thermal annealing resulted in the highest C/O ratio, indicative of the highest degree of oxygen group removal. Moreover, the C/O ratio for aerogels frozen at −5 °C was almost two-fold higher than for those frozen at −196 °C, indicating that a slow freezing rate promotes more effective removal of solvent and oxygen groups.

The effect of the thermal annealing on the composition of aerogels was further evaluated by XPS analysis. The XPS analysis of GO precursors presented elsewhere [35] exhibited lower C/O ratios for GO_e_ than for GO. The C1s peak deconvoluted into three components in both cases, namely the carbon atoms in C=C/C-C bonds, those in C-OH/C-O-C bonds and those in COOH bonds, respectively, which contributed with 43.2%, 49.3% and 7.5% in the GO precursor, while in the GO_e_ their contribution was 33.6%, 59.1% and 7.3%, respectively. These results suggested improved oxidation degree in the GO_e_ precursor with respect to GO. Upon reduction by chemical, hydrothermal and thermal annealing, the oxygen content markedly decreased. For example, the XPS survey and C1s high-resolution spectrum of the r_2T_GO_e_ is presented in Figure 3c. A small signal appeared in the survey spectrum, indicating N content. The C1s peak was deconvoluted into 4 components located at 284.56, 285.63, 286.51 and 288.32 eV which were assigned to C=C/C-C, C-N bond in the amine, C-OH/C-O-C and COOH, respectively [40].

**Figure 4:**
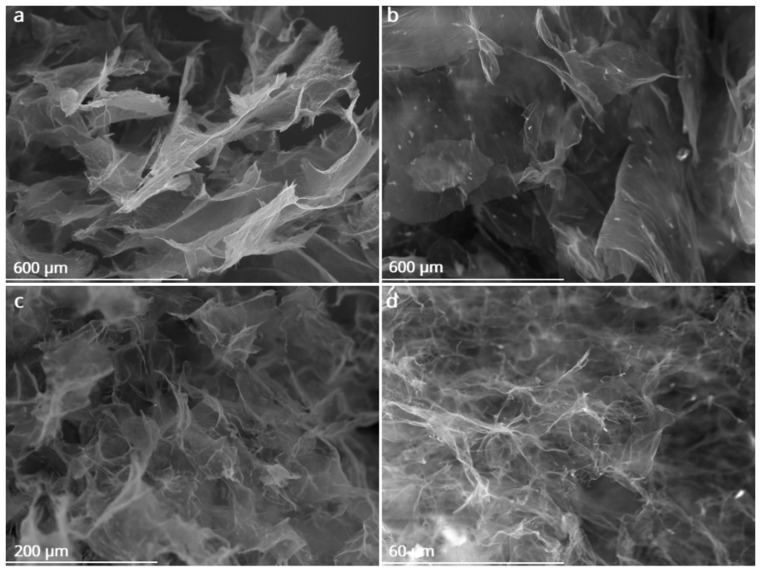
SEM images of r_1_GO (**left**) and r_1_GO_e_ (**right**) aerogels obtained by hydrothermal reduction (r_1_) freeze-cast at −5 °C (**a**,**b**) and freeze-cast at −196 °C (**c**,**d**).

The microstructure of the aerogels was examined using scanning electron microscopy (SEM), and the effect of hydrogel freezing temperature on the resulting aerogels was evaluated (at −5 °C and −196 °C). Figure 4a,b depicting SEM images of r_1_GO and r_1_GO_e_ subjected to freeze-casting at −5 °C, show large pores (~250 µm), resulting in microporous structures characterized by curled sheets in r_1_GO. The sheets observed in the case of aerogels obtained from GO_e_ exhibit a more transparent appearance (see r_1_GO_e_ in Figure 4b). In contrast, the aerogels made from hydrogels freeze-cast at −196 °C display compact structures with higher pore density and smaller pores, consistent with the formation of many ice nuclei and limited crystal growth (see Figure 4c,d), irrespective of the employed precursor.

**Figure 5:**
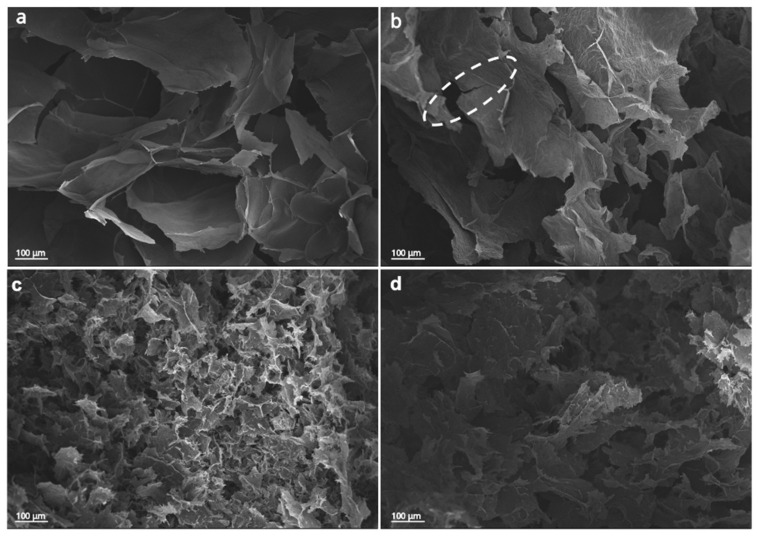
SEM images of (**a**,**b**) r_2T_GO_e_ and r_2T_GO aerogels freeze-cast at −5 °C and (**c**,**d**) r_2_GO_e_ and r_2T_GO_e_ aerogels freeze-cast at −196 °C (dashed box highlights the presence of fractures in the annealed aerogel with respect no non-annealed one).

As shown in Figure 5, depicting aerogels upon thermal treatment, the SEM microstructures revealed a porous network formed by sheets of reduced graphene oxide. This network was found to be loose with local voids and fissures compared to the unannealed network. This indicates a slight increase in porosity and more defined microchannels. The pore cavities in r_2T_GO_e_ freeze-cast at −5 °C are about 5 times larger than those produced by freeze-casting at −196 °C (see Figure 5c,d).

Raman, FT–IR, SEM, and EDS characterization techniques revealed changes in aerogels related to defect levels, reduction, and morphology, depending on the precursor, synthesis method, freeze-casting and annealing. This confirms the synergistic effect of such and points to a fine-tuning of each parameter.

### 3.2. Performance of the Engine Oil Sorbent

**Figure 6:**
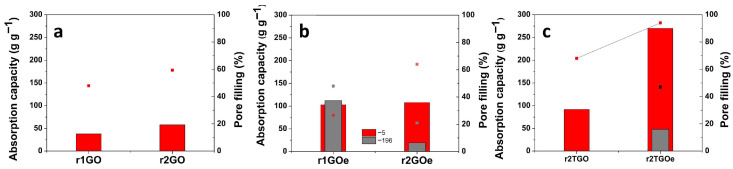
(**a**) Absorption capacity (bars) and pore filling (points) of GO aerogels freeze-cast at −5 °C; (**b**) GO_e_ aerogels freeze-cast at −5 °C and −196 °C; (**c**) aerogels obtained by hybrid reduction and thermal annealing.

The absorbency of aerogels is strongly influenced by several factors, including the reduction level, the structural properties of the precursor material, the freezing temperature (which affects nucleation and growth kinetics during freeze-casting), and changes that occur during annealing [41]. As shown in Figure 6a, for GO-based aerogels frozen at −5 °C, the combined reduction resulted in higher absorption capacity (56 g g^−1^) with respect to only hydrothermal reduction (36 g g^−1^), being accompanied by a similar trend in pore filling.

The GO_e_-based aerogels (see Figure 6b), under the same conditions, resulted in absorption capacities of similar values for both reduction approaches (about 105 g g^−1^). The filling percentages (color, above the bars) in this study exceeded previously reported values for GO-based aerogels, reaching about 65% by combining GO_e_ and the hybrid reduction approach. In the case of freeze-casting at −196 °C, r_1_GO_e_ aerogels showed a sorption capacity of 110 g g^−1^ with a filling of 47%. While the sorption capacity for r_1_GO_e_ aerogels did remain constant regardless of the freeze-casting temperature, the pore filling doubled when frozen at −196 °C, most likely due to more homogenous porosity. On the other hand, the r_2_GO_e_ freeze-cast at −196 °C showed an absorption capacity as low as 19 g g^−1^, which could be explained by the stronger removal of oxygen groups coupled with reduced dimensions of the pores that impede the absorption of pollutants. As shown in Figure 6c, the absorption capacities increased upon annealing to 91 g g^−1^ for r_2T_GO and 270 g g^−1^ for r_2T_GO_e_, with 94% pore filling observed in the latter case. In the r_3T_GO_e_ sample frozen at −196 °C, a sorption capacity of 50 g g^−1^ was recorded.

**Figure 7:**
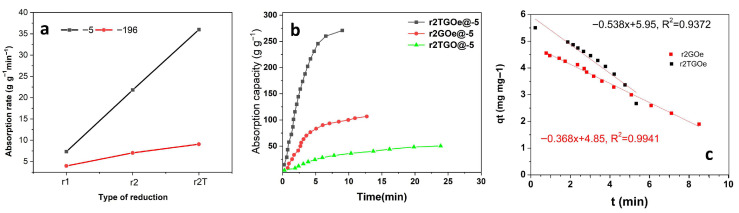
(**a**) Absorption rate of GO_e_ aerogels as a function of reduction approach and freeze-casting; (**b**) absorption kinetics of r_3_GO-based aerogels; (**c**) pseudo-first-order fitting for GO_e_ aerogels without and with thermal annealing.

Figure 7a shows the absorption rate for GO_e_ aerogels as a function of reduction approach and freeze-casting. It can be observed that an increasing trend is obtained by employing hybrid reduction and further thermal treatment for both freeze-casting rates. However, a marked increase is observed for the case of slow freezing, reaching about 20 g g^−1^ min^−1^ for r_2T_GO and approximately 30 g g^−1^ min^−1^ for r_2T_GO_e_. Figure 7b shows the kinetic profiles for the hybrid reduction approach and freeze-casting at a slow rate. It can be observed that the kinetic profile can be divided into two regions, with faster sorption in the first and limited capacity in the second. The time required to reach limiting value is the lowest for the hybrid reduction approach, GO_e_ and freeze-casting at −5 °C.

The absorption of the oil on the aerogels was evaluated by using the widely employed pseudo-first-order kinetic model due to its simplicity (see Figure 7c). This model is used to describe the sorption process between a solid sorbent and a liquid sorbate. The fitting results indicate a high correlation coefficient and a higher constant for the annealed and reduced aerogel with respect to the only reduced one. Pseudo-second-order and intraparticle models resulted in correlation coefficient values lower than 0.9, and thus these are not considered a good fit for the sorption mechanism.

## 4. Discussion

The findings confirm that the aerogel’s structure is influenced by multiple factors rather than a single one. These factors include the precursor material, the synthesis method, and the modeling of the porous structure during freeze-casting, as described for reduced graphene oxide (rGO) aerogels [23,40,41,42]. The effect of using an improved precursor—with a higher oxidation state and more oxygenated functional groups—was demonstrated by differences in the Raman and FT–IR spectra between graphene oxide (GO) and its modified version (GO_e_), supporting the use of expanded graphite [43]. More oxidation is known to provide more reactive sites for both reduction and cross-linking of graphene oxide nanosheets during hydrogel formation [44,45]. Essentially, the surface functional groups on graphene oxide nanosheets are key for creating aerogels [16,22]. This is evidenced by the lower ID/IG ratio seen in the r_1_GO_e_ aerogel compared to r_1_GO, as well as the microstructural features showing elongated nanosheets, which indicate the recovery of sp^2^ domains during the reduction of graphite oxide to graphene oxide. It also suggests a more uniform network with fewer defects when a precursor from expanded graphite is used after hydrothermal reduction [46].

The combined synthesis methodology results in higher C/O ratios and lower intensities in the FT-IR spectrum of oxygenated functional groups, consistent with observations from studies on the selective removal of these groups [30,47,48]. The partial restoration of sp^2^ domains enhances a more hydrophobic character and increases the aerogel’s affinity for organic–aromatic compounds [49,50]. In summary, incorporating vitamin C, which acts as a reduction-reinforcing agent, reproduces the behavior previously reported in other studies [22,28,51].

The freezing stage acts as a structural modulator, with the formation and growth of water crystals serving as a template that determines the size, shape, and connectivity of the pore network in the final aerogel. Freezing at −5 °C creates larger pores by removing larger solvent crystals and opening channels [24,44]. On the other hand, smaller solvent crystals result in smaller pores and more compact structures. This behavior is due to the physics of freeze-casting, in which the size of the ice crystals and the orientation of freezing determine the final structure of the aerogel [23]. The differences were substantial in GO_e_ aerogels due to the initial separation of the nanosheets.

Annealing treatment successfully removed the hydrophilic groups (O–H, C–O, C=O), thereby favoring the recovery of sp^2^-hybridized states and π–π stacking interactions in reduced graphene oxide nanosheets [52]. Consequently, this reinforces the hydrophobic and π–π interactions with the oil’s hydrocarbon chains. The vacancies and cracks observed in SEM of the heat-treated aerogels suggest that this may be due to the release of CO_2_ and H_2_O [28,53]. The XPS results emphasized the importance of the graphite precursor being tailored to the oxidation degree in GO. The contributions to C1s peak indicated improved oxidation degree in the GO_e_ precursor with respect to GO. Upon combined reduction and annealing, the contributions to C1s spectra in the corresponding aerogels, namely C=C/C-C (contributing with 78.1%), C-N bond in the amine (contributing with 12%), C-OH/C-O-C (contributing with 8.8%) and COOH (contributing with 1.1%), respectively [40], indicated a marked removal of oxygen groups along with functionalization with N due to nitrogen gas employed during annealing.

The increase in absorption capacities and kinetics observed is related to these synergistic freezing–annealing effects. The best configuration is found in r_2T_GO_b_ aerogels, with high hydrophobicity, low density, high porosity, and a more open three-dimensional architecture, values that far exceed most reported values, positioning it as a highly competitive material for oil recovery. These values are consistent with those reported in the literature for aerogels optimized by combined methods or doped with modifying agents [15,20,24,40,54]. The fitting of the process with the pseudo-first-order kinetic model suggests that the concentration of the oil is the main determinant of the sorption rate, as the aerogel porosity and high surface area offer many vacant sites to favor the sorption process. However, these aspects may need further investigation due to complex porosity and viscosity of oil sorbents.

A comparison with the available literature (Table 1) shows the performance of varying GO-based aerogels reduced with similar methods for absorption of oils. It is observed that the results enclosed in this work are in line with those reported for similar methods and analyzed oils. The results establish the following: (i) the precursor with the highest oxidation is ideal for producing more efficient aerogels; (ii) double reduction is effective for optimizing hydrophobicity; (iii) freezing at a lower temperature controls macrostructure and improves capillary transport; (iv) heat treatment further improves hydrophobicity and absorption capacity through structural purification. This comprehensive design route allows for easy modulation of engine oil sorbent performance.

## 5. Conclusions

In this study, a lightweight, high-performance reduced graphene oxide aerogel was developed using an integrated synthesis process based on the choice of precursor, reduction (hydrothermal–chemical), freeze-casting, and thermal annealing at 600 °C under nitrogen. Expanded graphite-based GO produced aerogels with uniform three-dimensional networks, low bulk density, and reduced oxygenated functional groups, resulting in a higher C/O ratio and greater motor oil absorption capacity than aerogels derived from a GO precursor. The combined effect of all parameters yielded an aerogel with enhanced hydrophobicity, more defined and accessible porosity for mass transport (greater penetration), and faster kinetics, all while remaining environmentally friendly. The obtained results indicated both reduction by removal of oxygen groups and nitrogen functionalization via a combined approach. The sorption process was found to fit with the pseudo-first-order kinetic model, as the pores and high surface area offered enough sites for the sorption, making the oil concentration the determinant of the sorption rate.

This study offers a comprehensive, transferable design criterion for manufacturing low-weight carbon-based sorbent materials for engine oil removal and environmental remediation. Future studies might explore surface functionalization and heteroatomic doping to improve performance and selectivity in hydrocarbons.

## Figures and Tables

**Table 1:** Comparison with other aerogels according to the reduction approach and sorbate.

Aerogel	Sorbate	Synthesis	Absorption Capacity	Ref.
rGO/Hydroxyapatite Nanowire (HAPNWs) aerogel	Oils, organic solvents	Chemical reduction	100–191 g g^−1^	[55]
Carbonized rGO/silk fibroin aerogel	Organic solvents, silicone oil, pump oil, gasoline, diesel	Chemical + hydrothermal reduction + heat treatment (Carbonized)	146.7–278.8 g g^−1^	[56]
Superhydrophobic graphene aerogel	Gasoline, diesel oil, engine oil, peanut oil, crude oil, organic solvents	Chemical reduction	109–236 g g^−1^	[57]
Enteromorpha-modified graphene aerogel	Engine oil, peanut oil, organic solvents	Hydrothermal reduction	68–200 g g^−1^	[15]
rGO composite aerogel	Organic solvents, soybean oil	Chemical + hydrothermal reduction	56.9–115.2 g g^−1^	[58]
Graphene aerogel (GA)	Organic solvents	Chemical reduction	240 g g^−1^	[59]
Anisotropic graphene aerogel	Organic solvents, vegetable oil, pump oil	Chemical reduction	120–200 g g^−1^	[60]
Graphene aerogels	Organic solvents	Chemical + hydrothermal reduction	105–160 g g^−1^	[22]
Functionalized graphene aerogels	Organic solvents	Chemical + hydrothermal reduction	48–96 g g^−1^	[49]
Robust reduced graphene aerogel	Engine oil	Hydrothermal reduction + heat treatment (calcined)	26 g g^−1^	[61]
rGO aerogel	Engine oil	Chemical + hydrothermal reduction + heat treatment	270.8 g g^−1^	This work

## Data Availability

The original contributions presented in this study are included in the article. Further inquiries can be directed to the corresponding author.

## Abbreviations

The following abbreviations are used in this manuscript:

GO	Graphene oxide
GO_b_	Highly oxidized graphene oxide obtained from expanded graphite
rGO	Reduced graphene oxide
r_1_GO	Aerogel obtained by hydrothermal reduction from GO only
r_1_GO_e_	Aerogel obtained by hydrothermal reduction from GO_b_ only
r_2_GO	Aerogel obtained by combined hydrothermal–chemical reduction from GO
r_2_GO_e_	Aerogel obtained by combined hydrothermal–chemical reduction from GO_b_
r_2T_GO	r_3_GO aerogel after post-synthesis thermal annealing
r_2T_GO_e_	r_3_GO_b_ aerogel after post-synthesis thermal annealing
FT-IR	Fourier transform infrared spectroscopy
SEM	Scanning electron microscopy
EDS	Energy-dispersive X-ray spectroscopy
